# Syringe Paper-Based Analytical Device for Thiamazole Detection by Hedysarum Polysaccharides-Mediated Silver Nanoparticles

**DOI:** 10.3390/mi14020350

**Published:** 2023-01-30

**Authors:** Dan Liu, Jiahui Ji, Xinran Guo, Sanhu Gou, Xinyue Chen

**Affiliations:** School of Pharmacy, Lanzhou University, Lanzhou 730000, China

**Keywords:** analytical device, thiamazole, silver nanoparticles

## Abstract

In this paper, silver nanoparticles (AgNPs) were successfully green-synthesized for the first time using Hedysarum polysaccharide (HPS) as a reducing agent, stabilizer, and modifier (HPS-AgNP). Thiamazole could induce the aggregation of HPS-AgNPs in the residue on a cellulose membrane. A syringe paper-based analytical device was creatively established to ensure the tightness, stability, and good repeatability of the test. The color information remaining on the cellulose membrane was converted into gray values using ImageJ software. Hence, the linear regression curve for thiamazole was established as y = 1 + 0.179x with a detection limit (LOD) of 24.6 nM in the relatively wide range of 0.1~10 μM. This syringe paper-based analytical device was successfully applied to the biological samples.

## 1. Introduction

Thiamazole, also known as tapazole, is universally used in the treatment of hyperthyroidism, which is the second most common endocrine system disease in humans nowadays [1,2,3,4,5]. It is necessary to note the importance and imminence of rational drugs that have become the common responsibility of the medical field. Especially for thiamazole, its narrow therapeutic window greatly limits the rationality and safety of its use. An excessive dose of thiamazole may lead to nausea, dizziness, diarrhea, and even nephritis, lupus erythematosus syndrome, etc. Some typical methods are reported and confirmed available for the detection of thiamazole, including flow-injection spectrophotometry, liquid chromatography-tandem mass spectrometry (LC-MS), enzyme multiplied immunoassay technique (EMIT), etc. [2,6,7,8,9]. These methods exhibit high accuracy and good sensitivity. However, they have inevitable drawbacks, such as being time-consuming and unable to realize the real-time detection that may cause serious consequences due to the failure of timely detection. Hence, a simple, rapid, and time-saving analytical method for thiamazole detection is in great need [10,11,12,13].

Paper-based analytical devices (PADs) were introduced by Whiteside’s group in 2007, the availability of which opened up new applications in the field of analytical devices. With the fast development in portable and disposable sensor technology [6,14,15], PADs have attracted significant attention from researchers all over the world. Starting from the original intention of medical diagnosis, the application of PADs soon expanded to various fields [16,17] that have been successfully applied to a variety of targets, including metals, medicine, pesticides, etc. Compared with other common analytical methods, PADs are highly competitive, owing to the advantages of simple operation, visuality, and fast readout. As one of the most commonly used materials in PADs [18,19], cellulose membrane has the properties of uniform pore size, large surface area, and high mechanical strength and is widely used as assay substrate. However, there are no reports on the successful construction of PADs for thiamazole assay. In addition, the previous PADs in colorimetric detection are mostly exposed directly to air or harsh environments, resulting in the poor precision and poor stability of the device. Hence, it is of high practical value to develop an enclosed PAD for thiamazole testing, improving the accuracy of detection and avoiding the interference of external conditions.

Due to the unique surface plasmon resonance (SPR) properties, silver nanoparticles (AgNPs) have been widely used in analysis and testing [20,21,22,23]. In this article, AgNPs were innovatively used as the substrates for PADs, owing to the advantage of small size, visibility, and sensitivity [9]. A variety of physical or chemical methods are generally used to prepare AgNPs. However, most of these methods inevitably involve the use of hazardous, toxic chemicals which may pose a potential risk to the environment or human health [18,24]. Thus, many researchers are focusing on green synthesis by using extracts of natural plants such as Phoenix dactylifera, Tridax procumbens, Limonia acidissima trees, Black rice, Murraya koenigii, etc. [25,26,27,28,29], which have the advantages of being green, clean, nontoxic and low priced. Moreover, plant extracts can simultaneously act in the roles of reducer, modifier, and stabilizer, realizing the rapid one-pot method synthesis [30,31,32,33]. Our group has been devoted to the study of Radix *Hedysari*, a traditional Chinese medicine that contains many active ingredients. Hedysarum polysaccharides (HPS) have been proven to be abundant in Radix *Hedysari*, which has good reducibility owing to the rich hydroxyl and aldehyde groups [34]. Our group has been committed to HPS research for a long time. Unlike this article [35], the monosaccharide composition of HPS was verified as glucose (Glu), arabinose (Ara), rhamnose (Rha), galactose (Gla), and mannose (Man). It provided a basis for the subsequent investigation of the reaction mechanism. Meanwhile, HPS was identified as the reducing polysaccharide, which can reduce metal ions to metallic elements. The content of hydroxyl in polysaccharide is very high, so reducing polysaccharides has certain chemical activity. Hence, HPS was considered to green-synthesize AgNPs, which could be well-used as substrate in PADs.

In this article, a syringe paper-based detection device using green-synthesized HPS-AgNPs as substrate was established for thiamazole testing. Thiamazole could trigger the aggregation of HPS-AgNPs, resulting in the formation of large-scale aggregates that easily accrues as residue on a cellulose membrane. Using ImageJ software, colorimetric information on the membrane was converted into gray values. Thus, a linear regression was established between the gray values and the thiamazole content and successfully applied to serum and urine samples. As with PADs, our syringe-detection device was proven to be tight, stable, and had a fast, instant readout.

## 2. Experimental Methods

### 2.1. Chemicals and Reagents

The dried roots of Radix Hedysari were purchased from a local pharmacy in Longnan, Gansu Province. Silver nitrate (AgNO_3_) was purchased from Sigma-Aldrich. CAM membrane and reusable filter paper holders were purchased from Ruihua Technology Service Co., Ltd. (Guangzhou, China). The standard of Roxithromycin, Lincomycin, Ofloxacin, Paracetamol, Salicylic acid, and Phenol were purchased from Aladdin Bio-Chem Technology Co., Ltd. (Shanghai, China).

### 2.2. Preparation of HPS

HPS was a crude polysaccharide prepared according to previous work using a combination of the enzyme complex and ultrasonic extraction process. Briefly, the dried Radix Hedysari root was ground into powder and degreased. Then, it was hydrolyzed with a complex enzyme and extracted by ultrasonication. The amount of compound enzyme added was 1.0% of the mass of HPS. The enzymatic hydrolysis temperature was 45 °C and the time was 1 h. The sonication temperature, power and time were 60 °C, 40 W, and 30 min, respectively.

### 2.3. Preparation of HPS-AgNPs

A certain volume of HPS (10 g/L) was gradually added to the AgNO_3_ solution (20 mL, 0.25 mM). The reactant was kept at 70 °C and magnetically stirred for 1 h. The color of the reaction solution gradually turned to light yellow. HPS-AgNPs were then collected and stored in the refrigerator at 4 °C before use.

### 2.4. The Optimization of External Conditions

The amount of reducing agent had a great impact on the particle size or morphology of the nanoparticles. To improve the analytical merit of the HPS-AgNPs, the volume of HPS (1 g/L) was changed from 50 μL to 2000 μL. The obtained HPS-AgNPs were used as detection substrates to ensure their suitability for the syringe paper-based device.

### 2.5. Characterization of HPS-AgNPs

The optical property of the HPS-AgNPs in the range of 300 nm to 800 nm was determined by a UV-visible spectrophotometer (UV-vis, Perkin Elmer, Waltham, MA, USA). Size distribution and the electric potential of the HPS-AgNPs were measured by employing the laser scattering dynamic instrument Zeta sizer Nano 3600 (DLS, Malvern, UK). The nanoparticles were pictured by Transmission electron microscopy FEI Tecnai G2TF20 instrument (TEM, FEI NanoPorts, Hillsboro, OR, USA). Meanwhile, the result of Energy disperses spectroscopy (EDS) was also obtained.

### 2.6. The Stability of HPS-AgNPs

The obtained HPS-AgNPs, which were synthesized at optimal conditions, were changed to different temperatures and pH values to check the effect of pH on them. The pH value of the colloid was adjusted from 1 to 14. The UV spectra of the samples were analyzed by a UV spectrophotometer, and the fluorescence color was recorded by a 365 nm UV lamp.

### 2.7. Design of the Syringe Paper-Based Device and Detection Method

As shown in figure in Section 3.4, the detection device consisted of 3 parts: a 2 mL syringe, a piece of CAM filter paper, and a reusable plastic filter holder. For colorimetric detection, a 50 μL sample was added to 450 μL of HPS-AgNPs, which was inhaled into the syringe beforehand. The solution was mixed thoroughly and allowed to stand for 1 min. Then, the mixture was introduced into the filter holder and passed through the CAM filter paper by pushing the peristaltic pump at a flow rate of about 1 mL/min. After all the liquid drained away, the CAM filter paper was removed by a tweezer and allowed to dry naturally. The desiccated CAM filter paper was placed on a white background and pictured using a smartphone. The color intensity of the CAM detection zone was converted to gray intensity using the image processing of ImageJ.

### 2.8. Interference Study

Possible antibiotic materials such as drugs, carbohydrates, and other interfering substances (ampicillin, clarithromycin, erythromycin, acetaminophen, starch, glucose, fructose, sucrose, maltose, sorbitol, lactose, 100 μM) that might interfere with biological samples were included as test subjects. These substances were all detected by using HPS-AgNPs, and the picture or UV-vis spectra results were recorded.

### 2.9. Real Sample Detection

Based on previous experiments, HPS-AgNPs were synthesized at optimal conditions. Human blood and urine samples were collected from healthy volunteers at the School of Pharmacy, Lanzhou University, China. An appropriate amount of acetonitrile solution was added to the human blood sample and then centrifuged for deproteinization and diluted 10 times. A human blood sample was set to stand for several hours to separate serum. A measure of 0.5 mL of serum sample was added with 5% (v:v) perchloric acid solution for deproteinization and then centrifuged at 8000 r/min for 10 min, and the supernatant was collected. A certain volume of NaOH (0.1 M) was added to adjust the serum to neutrality. The pretreated serum solution was diluted 5-fold and filtered through a 0.22 μm filter before use. Urine samples were also processed according to the above method. In accordance with the above detection method, a several concentrations of thiamazole (0.1, 1.0, 3.0, 5.0, 7.5, and 1.0 mM) were tested. 

## 3. Results and Discussion

### 3.1. Synthesis and Optimization of HPS-AgNPs

**Figure 1:**
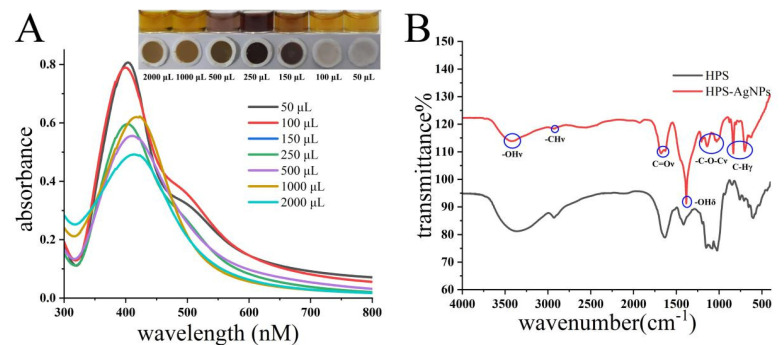
(**A**) The photographs and UV spectra of AgNPs with different doses of HPS. (**B**) The FT-IR spectra of HPS and HPS-AgNPs.

In the green synthesis, HPS played multiple roles as a reducing agent, modifier, and stabilizer. The initial pH of the reaction solution was 12, and the concentration of AgNO_3_ was 0.25 mM. Different doses of HPS solution were added to the reaction solution. The reaction system was heated to 70 °C under magnetic stirring for 1 h. The color of the solution was observed to gradually deepen with the increase in reaction time. As shown in Figure 1A, the HPS-AgNPs appeared yellow in color when the dose of HPS was 50 μL or 100 μL. At a dose of 250 μL of HPS, the HPS-AgNPs were brown in color. The results showed that the peak shape and intensity of the UV absorption peaks of the HPS-AgNPs changed significantly with the dose of HPS. When the added HPS was 50 μL or 100 μL, a smooth UV absorption spectrum appeared with a high characteristic UV absorption peak. However, when the dose of HPS exceeded 150 μL, a smooth but less-intense single peak appeared near the 400 nm wavelength. Moreover, as the dose of HPS increased, the absorption peak red-shifted and a shoulder peak appeared at 500 nm, suggesting the formation of large-sized nanoparticles. To select suitable HPS-AgNPs for subsequent syringe paper-based assays, different-sized HPS-AgNPs were tested. As shown in Figure 1, when the dose of HPS was 100 μL, the obtained HPS-AgNPs had almost no color residue on the CAM filter paper. However, as the dose exceeded 150 μL, the synthesized HPS-AgNPs remained on the dark-colored CAM filter paper. Since the dark color of the CAM filter paper would affect the result of colorimetric assay, it was not considered to be a suitable PAD substrate. Hence, 100 μL HPS (1 g/L) was selected as the optimal reducing agent for further experimentation.

### 3.2. Characterization of HPS-AgNPs

The FT-IR spectra of HPS and the HPS-AgNPs were compared (Figure 1B). The C-H stretching vibration (2958–2927 cm^−1^), C-H out-of-plane bending vibration (600–1000 cm^−1^), O-H stretching vibration (3700–3200 cm^−1^), O-H bending vibration (1410–1260 cm^−1^), and C-O-C stretching vibration (1270–900 cm^−1^) peaks could be found in the FT-IR spectra of HPS. Compared with HPS-AgNPs, characteristic peaks at almost the same place could be found, indicating that HPS participated during the synthesis process of HPS-AgNPs and was successfully modified on the surface of the nanospheres.

**Figure 2:**
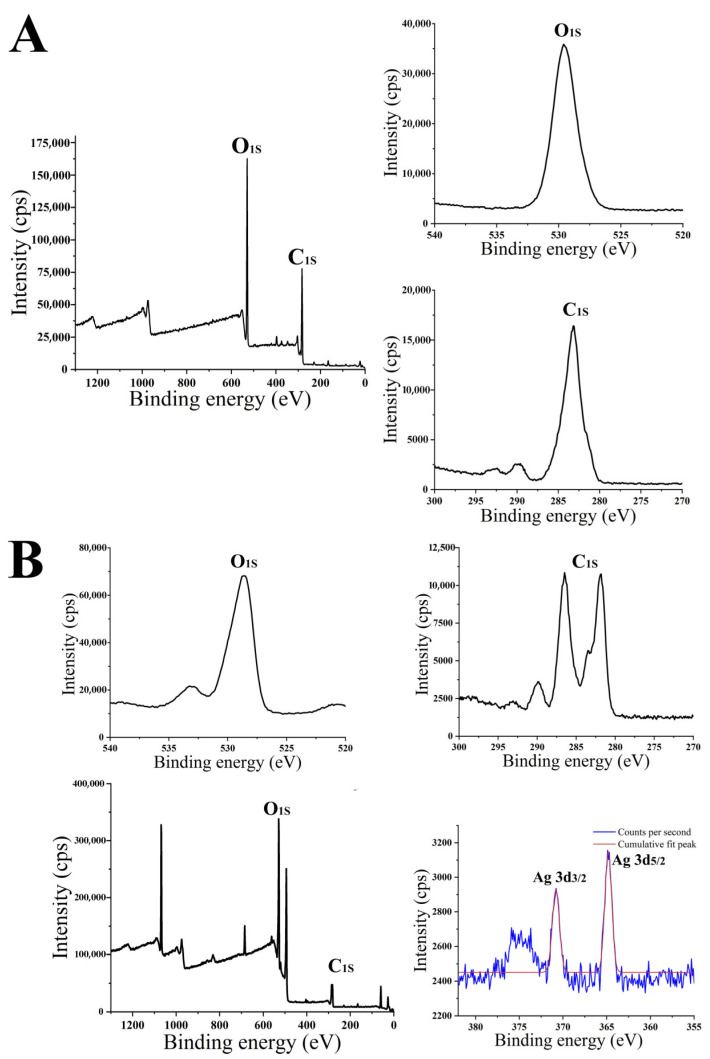
The XPS results of (**A**) HPS and (**B**) HPS-AgNPs.

HPS-AgNPs and HPS were also analyzed by XPS. As shown in Figure 2, the peaks of 528.54 eV (1 s)/280~290 eV (1 s) appearing in the spectra of the HPS-AgNPs could be found at almost the same position in the spectra of the HPSs, associating with oxygen/carbon, respectively. The typical peaks of Ag 3d (364.9, 370.9 eV) were associated with the Ag 3d_3/2_ binding energy and Ag 3d_5/2_ binding energy, respectively. The peaks for silver atoms typically occurred at 368.2 eV and 374.3 eV. It should be noted that due to the presence of HPS, the interaction between HPS and the silver atoms resulted in a slight shift of the peaks.

### 3.3. The Stability of HPS-AgNPs

**Figure 3:**
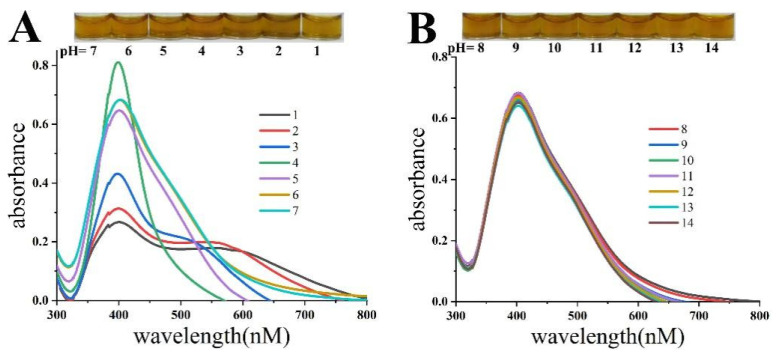
The photographs and UV spectra of HPS-AgNPs under (**A**) acidic and (**B**) basic conditions.

The influence of acidic or alkaline conditions on the HPS-AgNP colloid was examined, as shown in Figure 3. When the pH varied from 4 to 1, the absorbance intensity of the HPS-AgNPs decreased, while the original single sharp SPR peak developed into a broad double peak. This proved that the HPS-AgNPs were unstable under acidic conditions, accompanied by the black precipitates deposited at the bottom of the vessel. It was suggested that the positively charged hydrogen ions in the acidic environment disrupted the electrostatic state of the nanoparticles, leading to a massive aggregation of HPS-AgNPs. However, when the pH was increased to four, the absorption intensity of the HPS-AgNPs significantly increased with a yellow color. When the pH value of the HPS-AgNP colloid was adjusted from 5 to 7, it could be observed that the color or characteristic absorption of the HPS-AgNPs remained stable. In alkaline conditions (pH 8–14), the spectrum or color characteristics of the HPS-AgNPs were almost unchanged. Thus, it could be inferred that the HPS-AgNPs remained stable in pH values ranging from 5 to 14.

### 3.4. Detection Method of Thiamazole

**Figure 4:**
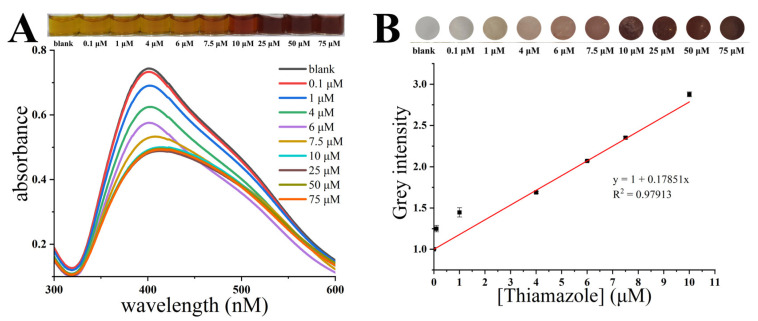
(**A**) The photograph and UV-vis spectra of the HPS-AgNP detection results of methimazole. (**B**) The results and linear diagram of the detection results of thiamazole by the syringe paper-based detection device.

**Figure 5:**
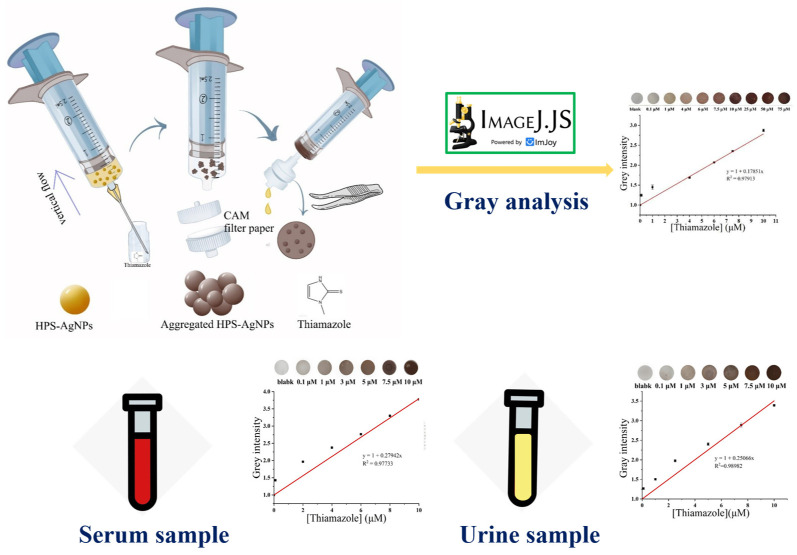
The flow diagram of the experimental activities of the syringe paper-based detection device and detection method.

The photographs and UV–vis spectra of the HPS-AgNPs added with different concentrations of thiamazole are shown in Figure 4A. For the uniformly dispersed HPS-AgNP colloid, a maximum UV absorption at 401 nm was observed due to the SPR effect. With the increase in thiamazole concentration from 0 to 10 μM, the maximum UV absorption value gradually increased, with a slight red shift from 401 nm to 412 nm. The shape of the SPR peak also changed from sharp to smooth, accompanied by a change in the color of the solution from yellow to brown. It was indicated that thiamazole induced the aggregation of the HPS-AgNPs. However, when the thiamazole concentration exceeded 10 μM, the UV absorption or the color of the solution no longer showed significant changes, indicating that the aggregation state of the HPS-AgNPs reached saturation and the addition of more thiamazole could no longer induce further aggregation of HPS-AgNPs. Such large-sized aggregations were easily retained by the small pore-sized CAM filter paper, leaving an imprint of a certain color on the CAM filter paper. The HPS-AgNPs were applied to the syringe paper-based detection device to further evaluate the analytical merit of the device. Figure 5 clearly illustrates the flow diagram of the experimental activities of the syringe paper-based detector for thiamazole detection. Briefly, this device consisted of three parts: a 2.5 mL syringe, a reusable filter holder, and a circular cellulose acetate CAM filter paper with a diameter of 10 mm. The colorimetric assay procedure was as follows: Different concentrations of thiamazole solution were added to the syringe with the HPS-AgNP solution pre-inhaled. The syringe was shaken well and mixed for 1 min. Then, the syringe was pushed to pass the solution through the CAM filter paper. Finally, the CAM filter paper was removed and dried naturally. According to the established detection method, the thiamazole concentration in the range of 0–75 μM was detected. The results are shown in Figure 4B. It could be seen that there were no obvious residues on the detection CAM filter paper, indicating that the dispersed HPS-AgNPs could pass through the CAM filter paper without leaving residues. With the increase of thiamazole concentration, the color on the CAM filter paper gradually deepened. It could be visibly found that the color deposited on the CAM filter paper was positively related to the concentration of thiamazole. The software ImageJ was used to convert the color information into a gray value, establishing a linear relation between the thiamazole concentration and the gray value. Within the range of 0–10 μM, the regression equation was calculated to be y = 1 + 0.179x with a LOD value of 24.6 nM and R^2^ of 0.979 (LOD = 3 σ/s, where σ represents the standard deviation of the blank and s represents the slope of the linear regression equation).

HPS-AgNPs were extensively characterized in the absence or presence of thiamazole to further illustrate the reaction mechanism. As shown in Figure S1, in the absence of thiamazole, the HPS-AgNPs remained uniformly dispersed, with a measured average particle size of 8.51 ± 1.89 nm, whereas after the addition of thiamazole, the HPS-AgNPs rapidly aggregated when the particle size increased to 212.58 ± 51.53 nm. In addition, the Energy-dispersive X-ray spectroscopy attached to the TEM device further demonstrated the abundant Ag atoms existing in the HPS-AgNP sample to be tested. As measured by laser dynamic light scattering (Figure S2), the average particle size of the dispersed HPS-AgNPs was 7.63 ± 2.50 nm. With the addition of thiamazole, the average particle size of the HPS-AgNP aggregation was 188.63 ± 149.04 nm. The results obtained by TEM were basically consistent with the results of DLS, confirming the crosslink reaction between the HPS-AgNPs and the target of thiamazole.

### 3.5. Selectivity of Thiamazole by HPS-AgNPs

**Figure 6:**
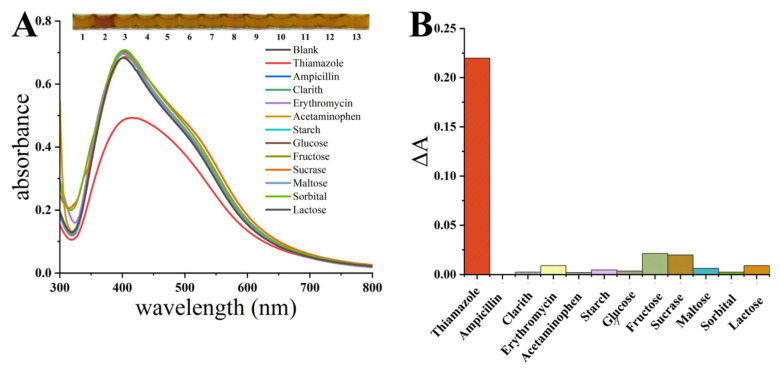
(**A**) The UV-vis spectra of HPS-AgNPs in the presence of different targets; (**B**) the histogram of absorbance in the presence of different targets corresponding to the spectra (**A**).

Various interferents (thiamazole, ampicillin, clarith, erythromycin, paracetamol, amylum, glucose, fructose, invertase, maltose, sorbitol, lactose) generally existing in biological samples were tested with a final concentration of 100 μM. To further confirm the good selectivity of thiamazole, the final concentration of thiamazole was determined to be 10 μM. As shown in Figure 6A, only thiamazole caused a significant decrease in the UV-vis absorption peak of the HPS-AgNP solution, accompanied by the color varying from yellow to brown. The addition of other substances caused no obvious change, indicating that these various interfering substances could hardly affect the detection of thiamazole. A histogram was established using the difference in absorbance between the sample and the blank control at 400 nm (∆A). Figure 6B shows the huge difference between the thiamazole and the other interfering substances. The above results confirmed that the HPS-AgNPs had a high selectivity for thiamazole testing, which supports the great potential for their application in biological samples.

### 3.6. Colorimetric Sensing of Thiamazole in Serum and Urine Samples

**Figure 7:**
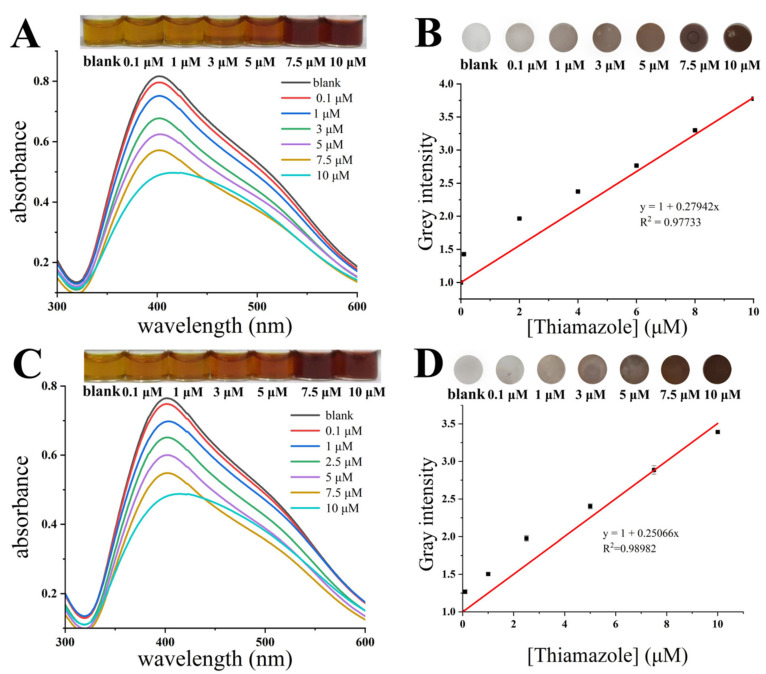
HPS-AgNPs detection of methimazole in (**A**) serum samples and (**B**) urine samples result in photos and UV spectra. Syringe paper-based detection device for methimazole in (**C**) serum samples and (**D**) urine samples result in photos and standard curves.

To ensure the use of HPS-AgNPs in medical applications, thiamazole was spiked in the serum and urine samples and measured using the syringe paper-based detection device. As shown in Figure 7A, the characteristic SPR peak of the HPS-AgNPs appeared at 401 nm with the addition of a blank human serum sample. As the concentration of thiamazole ranged from 0.1 μM to 10 μM, the absorption intensity of the HPS-AgNPs at 401 nm gradually decreased along with the changed color from yellow to brown, indicating the aggregation of HPS-AgNPs. The correlation between the concentration of thiamazole and the gray intensity was positively related: y = 1 + 0.0279x, R^2^ = 0.977 with a LOD of 65.3 nM (Figure 7B). The same experiments were performed on spiked urine samples, as shown in Figure 7C,D. The linear equation between the concentration of thiamazole and the gray intensity was also established: y = 1 + 0.0251x, R^2^ = 0.989 with a LOD of 52.6 nM (LOD = 3 σ/s, where σ represents the standard deviation of the blank, and s represents the slope of the linear regression equation.).

## 4. Conclusions

In conclusion, a novel AgNPs reduced and modified by HPS (HPS-AgNPs) and a syringe paper-based detection device constructed with HPS-AgNPs as the detection substrate for methimazole detection were developed for the first time based on the good reducing properties of HPS. The experimental results showed that the HPS-AgNPs had a sensitive and highly selective specific agglomerative response to methidazole. Correspondingly, the syringe paper-based assay device also showed good performance for the detection of methimazole and a low LOD of 24.6 nM. The device was low-cost, easy to operate, sensitive, specific, and had been successfully used for the naked-eye colorimetric detection of methimazole in real human serum and urine samples. In real-life scenarios, the quantitative analysis of the colorimetric results can be achieved by visual observation only or through the combined use of smartphones and computer software.

## Data Availability

Data available on request due to restrictions e.g., privacy or ethical. The data presented in this study are available on request from the corresponding author.

## Supplementary Materials

The following supporting information can be downloaded at: https://www.mdpi.com/article/10.3390/mi14020350/s1, Figure S1: The electron micrograph of (**A**) dispersed HPS-AgNPs; (**B**) HPS-AgNPs in the presence of thiamazole; (**C**) EDS spectroscopic analysis results of HPS-AgNPs. Figure S2: The DLS results of (**A**) dispersed HPS-AgNPs and (**B**) HPS-AgNPs in the presence of thiamazole.
