# Supplementary material for: Syringe Paper-Based Analytical Device for Thiamazole Detection by Hedysarum Polysaccharides-Mediated Silver Nanoparticles
Source: Micromachines (Basel). 2023 Jan 30;14(2):350. doi: 10.3390/mi14020350 (PMC9962882; doi:10.3390/mi14020350)
Supplement: Supplementary file 1 [file micromachines-14-00350-s001.zip › micromachines-2185718-supplementary.pdf]

Syringe paper-based analytical device for thiamazole detection by Hedysarum polysaccharides-mediated silver nanoparticles

Dan Liu[#], Xinran Guo[#], Sanhu Gou and Xinyue Chen[1*]

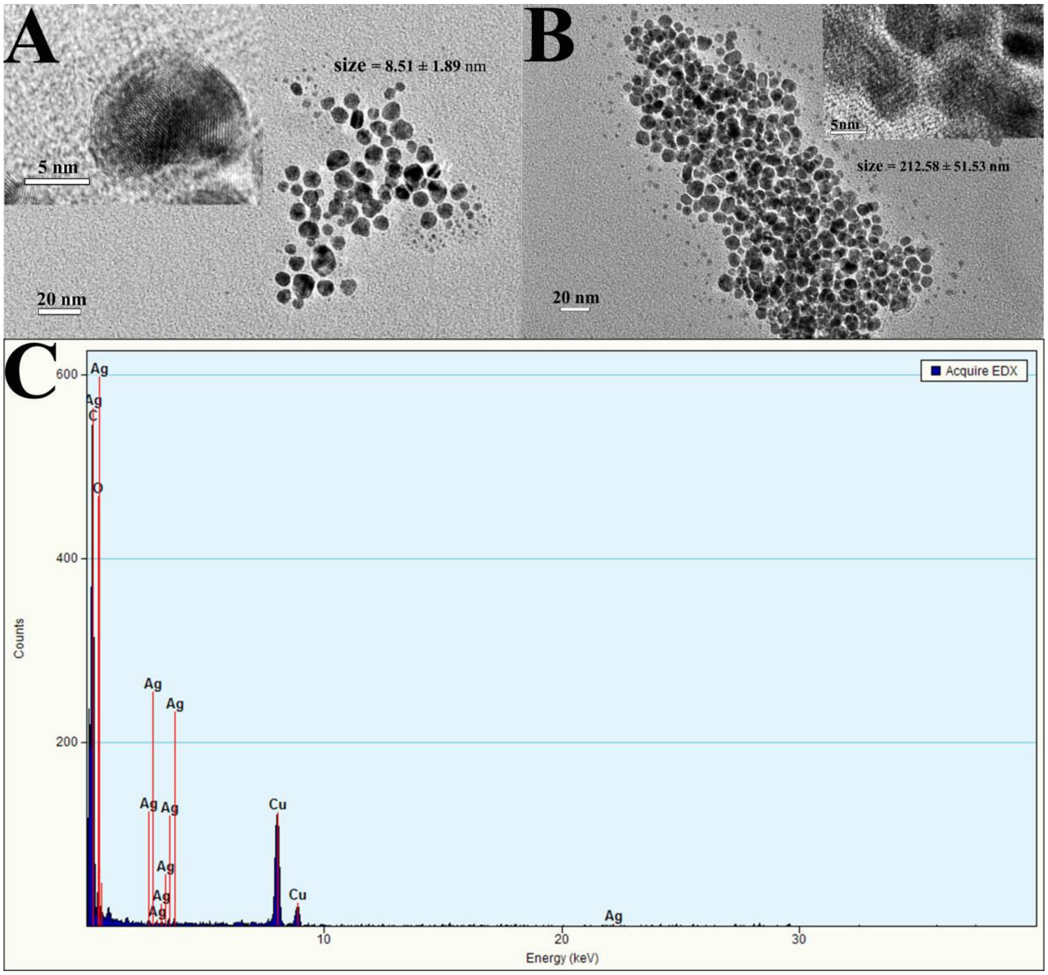

Figure S1. The electron micrograph of (A) dispersed HPS-AgNPs; (B) HPS-AgNPs in the presence of thiamazole; (C) EDS spectroscopic analysis results of HPS-AgNPs.

[#] These authors contributed equally to the article.

* School of Pharmacy, Lanzhou University, Lanzhou, 730000, P. R. China. E-mail: chenxinyue888@126.com; Tel: (+86) 15293109642

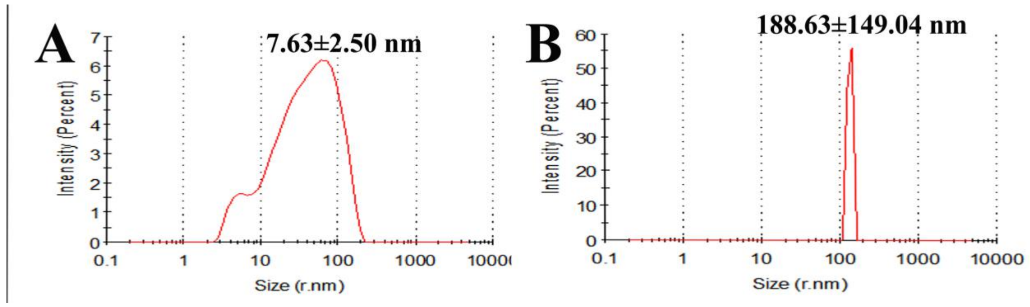

Figure S2. The DLS results of (A) dispersed HPS-AgNPs and (B) HPS-AgNPs in the presence of thiamazole
